# Prevalence of childbirth fear in an Australian sample of pregnant women

**DOI:** 10.1186/1471-2393-14-275

**Published:** 2014-08-14

**Authors:** Jocelyn Toohill, Jennifer Fenwick, Jenny Gamble, Debra K Creedy

**Affiliations:** School of Nursing & Midwifery, Griffith University, Logan Campus, University Drive, Meadowbrook, QLD 4131 Australia; Gold Coast University Hospital, 1 Hospital Blvd, Southport, QLD 4215 Australia

**Keywords:** Childbirth fear, Prevalence, Pregnancy, WDEQ-A, Caesarean section, Parity

## Abstract

**Background:**

Childbirth fear is reported to affect around 20% of women. However reporting on levels of symptom severity vary. Unlike Scandinavian countries, there has been limited focus on childbirth fear in Australia. The aim of this paper is to determine the prevalence of low, moderate, high and severe levels of childbirth fear in a large representative sample of pregnant women drawn from a large randomised controlled trial and identify demographic and obstetric characteristics associated with childbirth fear.

**Method:**

Using a descriptive cross-sectional design, 1,410 women in their second trimester were recruited from one of three public hospitals in south-east Queensland. Participants were screened for childbirth fear using the Wijma Delivery Expectancy/Experience Questionnaire Version A (WDEQ-A). Associations of demographic and obstetric factors and levels of childbirth fear between nulliparous and multiparous women were investigated.

**Results:**

Prevalence of childbirth fear was 24% overall, with 31.5% of nulliparous women reporting high levels of fear (score ≥66 on the WDEQ-A) compared to 18% of multiparous women. Childbirth fear was associated with paid employment, parity, and mode of last birth, with higher levels of fear in first time mothers (p < 0.001) and in women who had previously had an operative birth (p < 0.001).

**Conclusion:**

Prevalence of childbirth fear in Australian women was comparable to international rates. Significant factors associated with childbirth fear were being in paid employment, and obstetric characteristics such as parity and birth mode in the previous pregnancy. First time mothers had higher levels of fear than women who had birthed before. A previous operative birth was fear provoking. Experiencing a previous normal birth was protective of childbirth fear.

## Introduction

Childbirth is a significant physiological, social and emotional event in the life of a woman and her family. Although inherently unpredictable, the experience of childbirth should be life-affirming and associated with minimal risk of an adverse outcome for women living in developed countries [1]. Unfortunately, childbirth has become increasingly medicalised and risk adverse [2, 3]. Although the reasons for this are complex, there is increasing evidence that childbirth fear [4–6] stemming from birth and non-birth related factors contribute to longer labour and higher caesarean section (CS) rates [4, 7, 8]. Women experiencing long, painful, or medicalised births are more likely to report birth as traumatic and are at higher risk of postnatal depression [9, 10]. The extent and consequences of childbirth fear in the Australian population compared to international reports is limited. This paper reports on a descriptive cross-sectional study that aimed to determine the prevalence of childbirth fear in a large sample of Australian women and identify associated demographic and obstetric characteristics.

## Background

There is growing international interest about the role fear may play in women’s decisions about birth mode as well as of the contribution of interventions such as caesarean section to the development of fear symptoms (see for example, [11–19]). Research into childbirth fear has its origins in Scandinavian countries and commenced in earnest with the work of Areskog [20]. Subsequently this work led to development of the Wijma Delivery Expectancy/Experiences Questionnaire Version A (WDEQ-A) [21], which is now the most commonly used measure of childbirth fear.

### Current knowledge of prevalence of childbirth fear

Internationally, the prevalence of fear associated with childbirth is often reported to be around 20% with 6-10% of these women experiencing intense fear of labour and birth that impacts their daily activities (with intrusive thoughts or high anxiety) and ability to positively anticipate a normal birth [22, 23]. However prevalence rates of childbirth fear vary. Different measures and definitions of fear are likely to contribute to these results. Arguably cultural factors, parity, and timing of data collection are also likely to influence reported levels of childbirth fear.

When measured by the WDEQ-A [21], the prevalence of high levels of childbirth fear (score ≥66) has been consistently reported to be between 24% and 26% [for example, Johnson and Slade [14], Hall et al. [16], Zar, Wijma and Wijma [24] – also see Table 1].

**Table 1:** **Prevalence of childbirth fear by WDEQ-A cut points**

Author, Yr, Country	Gestation screened	WDEQ cut point	Population	Parity	Prevalence
Ryding, 1998. [5]. Sweden.	K32	WDEQ ≥85	Swedish	All	10%
				Nullips, Mean 54.1	
				Multis, Mean 57.7	
Zar, 2001. [24]. Sweden.	K32	WDEQ ≥66	Swedish	All, Mean 57	26%
				Nullips, Mean 58.6	26%
				Multis, Mean 50.3	26%
Johnson, 2002. [14]. UK.	K32	WDEQ -	UK	Nullips, Mean 65.4	-
				Multis, Mean 58	
Zar, 2002. [25]. Sweden.	K32	WDEQ ≥ 85	Swedish	All, Mean 57	11%
		WDEQ ≥ 100			2.4%
Kjergaard, 2008. [22]. Sweden.	K37	WDEQ ≥85	Swedish & Danish	Nullips, Mean 59	11%
Fenwick, 2009. [6]. Australia.	K36	WDEQ ≥ 66	Australian	All, Mean 57.8 (6–115)	27%
				Nullips, Mean 62.4	33%
				Multis, Mean 53.6	20%
Hall, 2009. [16]. Canada.	K35-39	WDEQ ≥66	Canadian	All, Mean 52.9	25%
Spice, 2009. [26]. Canada.	K24	WDEQ ≥85	Canadian	All	9.1%
Nieminen, 2009. [27]. Sweden.	K16-32	WDEQ ≥ 85	Swedish	All, Mean 62.8	15.6%
				Nullips, Mean 64.5	14.8%
				Multis, Mean 60.7	16.5%
		WDEQ ≥ 100		All, Mean 62.8	5.7%
Rouhe, 2009. [28]. Finland.	K22	WDEQ ≥ 100	Finnish	All, Mean 68.3	
				Nullips < K20, Mean 70.7	7.0%
				Nullips > K20, Mean 74.7	
				Multis < K20, Mean 63	7.7%
				Multis > K20 Mean 69.7	
Adams, 2012. [4]. Norway.	K32	WDEQ ≥85	Norwegian	All – Mean 56.7	7.5%
Nordeng, 2012. [29]. Norway.	K32	WDEQ ≥85	Norwegian	All	7.8%
Storksen, 2012. [30]. Norway.	K32	WDEQ ≥85	Norwegian	All – Mean 56.8	8%
Salomonsson, 2013. [31]. Sweden.	K26	WDEQ ≥85	Swedish	Nullips, Mean 68.5	20.8%
Rouhe, 2013. [32]. Finland.	K11-13	WDEQ ≥ 100	Finnish	Nullips	8.1%

In Australia, there has been limited work on the phenomena of childbirth fear [6, 33, 34]. One of the few studies to inform this issue was conducted in Western Australia. Using an explorative descriptive design, researchers found that around 30% of mothers (n = 202) used words such as *terrifying* and *petrifying* to describe their expectations of birth [35]. In a follow up study Fenwick et al. [6] used the WDEQ-A to measure childbirth fear in 400 pregnant women in their third trimester. These researchers found that around half the population recorded moderate fear levels (score of 38–65) with 25% recording high levels of childbirth fear (score ≥66) and only 25% recording low levels of fear (score ≤ 37) [6].

One other Australian study on childbirth fear was conducted by Haines et al. [33] who used a two-item visual analogue scale, known as the Fear of Childbirth Scale (FOBS), to measure childbirth fear. The researchers screened and compared Australian and Swedish rural women in their second trimester of pregnancy. Nearly 30% of Australian and Swedish women were identified as highly fearful of birth with significantly higher fear levels in Swedish primiparous women compared with multiparous, but there was no significant difference in parity for Australian women.

### Factors contributing to childbirth fear

Demographic and obstetric factors may play a major role in the development of women’s childbirth fears [36]. Studies investigating demographic factors contributing to childbirth fear have shown mixed results in relation to age [27, 33, 37]; education [31, 33, 37]; and employment status [8, 18, 31, 38]. Financial worries may contribute to fear. Some previous studies relied on education levels, employment status or household living space as proxy indicators of wealth [31, 32, 37], but few have asked explicitly about household income. In respect to ethnicity, one Swedish investigation found immigrant women had higher fear levels [18]. No relationship has been found between being married and fear [31, 39]. It could be that marital status per se is not a sensitive indicator and of greater interest may be measures of relationship stability, satisfaction, and support in the relationship.

Most studies have found women expecting their first baby to be more fearful than multiparous women although the reverse has also been demonstrated [5, 16, 24, 27, 28]. When parous women report fear, it is often a result of previous negative and/or traumatic birth experiences [23, 24, 40]. Although there are inconsistencies in the literature [14], some studies have shown that women who are fearful in pregnancy are more at risk of unplanned obstetric interventions such as caesarean section [5, 18, 27]. Swedish researchers (27) found evidence that childbirth fear was associated with unplanned or emergency caesarean. Sometime later Australian researchers found high fear was significantly associated with high epidural rates and caesarean section [6]. Ryding and colleagues [41] recently confirmed significant risk of caesarean in fearful multiparous women in a cohort of over 7,000 Swedish women. Women experiencing caesarean section and instrumental birth have also reported increased fear in subsequent pregnancies [27, 42, 43].

Given the limited understanding of childbirth fear within the Australian context, it is opportune to determine the prevalence of childbirth fear in a large, representative sample of pregnant women. Our objectives were to determine:

1. The prevalence of low, moderate, high (WDEQ-A ≥66) and severe levels (WDEQ-A ≥85) of childbirth fear in a large representative sample of pregnant women; and
2. Identify demographic and obstetric characteristics associated with childbirth fear.

## Method

### Study context and recruitment

The data used in this study were collected as part of a multi-site randomised controlled trial (known as BELIEF) which tested the effects of a psycho-education intervention for high levels of childbirth fear compared to that of standard maternity care in south-east Queensland [44].

Little is known about when childbirth fear develops or the trajectory of fear across pregnancy. Therefore, pregnant women in their second trimester of pregnancy were invited to participate. Inclusion criteria included women ≤ 24 weeks gestation, aged 16 years and older, able to read, write and understand English and capacity to consent.

Women were recruited in the antenatal clinics of three south-east Queensland metropolitan teaching hospitals between May 2012 and June 2013. Approximately 8,500 publicly funded births occur within these hospitals annually.

A number of recruitment strategies were used. Firstly, all women booking-in at the participating sites received a study flyer by mail with their antenatal booking appointment and could therefore contact the researcher to register their interest in participating in the study. Secondly, midwives and midwifery students, trained to recruit women, attended antenatal clinics each day. All women meeting the study criteria were approached to participate including those who had already registered interest. In some instances women were identified as suitable for the study by hospital staff and referred to a research midwife. The research midwife confirmed that a woman met the inclusion criteria, explained participation in the study, provided an information sheet, and gained written consent. Ethics approval was obtained from Griffith University and multi-site approval for the three participating Queensland Health hospitals (Gold Coast, Logan and Redlands Hospitals) was received from the Gold Coast Health Service District Human Research Ethics Department.

A total of 2,311 eligible women were approached to participate in the study. Of these 61% (n = 1,410) were recruited. Reasons for non-participation are outlined in Table 2. Immediately following consent to participate, women completed a questionnaire that sought data about demographic characteristics (i.e., age, education, income) and obstetric history (i.e., parity, previous obstetric complications, and details of the previous birth mode) The Wijma Delivery Expectancy/Experience Questionnaire Version A (WDEQ-A) was used to measure childbirth fear.

**Table 2:** **Reasons for declining study participation**

Declined					
	Not interested*	Young children	Busy	Partner declined	Unwell
Study Site 1	225	18	8	1	0
Study Site 2	451	10	27	1	1
Study Site 3	141	4	14	0	0
Total	817	32	49	2	1

Not Interested *> 80% in company of close relative.

### Measure

The WDEQ-A measures fear of childbirth by asking women to rate their depth of feeling against 33 expectations and experiences before birth (version A) and after birth (version B) [21]. Questions are presented in positive and negative formats on a six-point Likert scale from 0–5 requiring reverse scoring of positively formulated questions. A score equal to or lower than 37 is considered low fear, a score between 38 and 65 equates to moderate fear and a score equal to or higher than 66 represents a high level of fear [24]. In this current study, the WDEQ-A was found to have a Cronbach’s alpha coefficient of 0.94.

### Data analysis

Frequencies were performed on all variables to ensure that minimum and maximum values were correct. Descriptive statistics were conducted on all variables. Total scores for all standardised measures were calculated. Analyses were firstly conducted for the whole sample and then within parity groups against characteristics of interest that may impact high childbirth fear levels. Tests for parametric data were: Independent T-test and Pearson’s product–moment correlation coefficient; and for non-parametric data tests were: chi square, Spearman’s Rank Order Correlation, Kruskal-Wallis and Mann–Whitney U.

## Results

### Sample characteristics

At recruitment, the majority of participants (n = 1400, 99%) were in their second trimester of pregnancy (Mean = 18.6 weeks, SD 2.96, range 11–25 weeks). The mean age of participating women was 28.8 years (SD 5.5, range 17–51 years). With regard to educational level some 80% of women (n = 1,131) had completed Year 12/Diploma level or achieved degree level or postgraduate education. The majority of women (n = 1,307, 92.8%) were in a relationship. Seventy-four percent of women were born in Australia, with just under 2% (n = 27) identifying as Aboriginal. The majority of women (n = 943, 67%) were in some form of paid employment with 36% (n = 506) employed full-time, 17% (n = 235) part-time and 14% (n = 202) in casual employment. Table 3 reports descriptive statistics for participant characteristics according to parity. The sample was comparable to state and national birthing populations.

**Table 3:** **Participant characteristics by parity compared to state and national birthing populations**

Characteristics	Nulliparous women	Multiparous women	Total sample	State birthing population	National birthing population
	n = 609	n = 801	n = 1,410	n = 61,020	n = 294,814
	n (%)	n (%)	n (%)	n (%)	n (%)
**Age** yrs Mean (SD, range)	28.8 yrs (5.2, 17 to 42)	30.1 yrs (5.2, 18 to 51)	28.8 yrs (5.5, 17 to 51)	29.2 yrs (ns, <20 to >40)^a^	30 yrs (ns,<15 to 60)^a^
Missing	-	1 ( 0.1)	1 (0.1)	-	74 (0.0)^a^
**Marital status**					
Single/divorced/separated	38 ( 6.2)	59 ( 7.4)	97 (6.8)	7,334 (11.9)^b^	-
Married/defacto/relationship	566 (93.0)	741 (92.5)	1307 (92.8)	52,834 (86.5)^b^	64% (All)^c^
Missing/Other	5 (0.8)	1 (0.1)	6 (0.4)	937 (1.5)^b^	
**Education**	606 (99.5)	800 (99.9)	1406 (99.7)	-	-
≤Year 12	307 (50.4)	395 (49.3)	702 (49.8)	-	-
Post Year 12	299 (49.1)	405 (50.6)	704 (49.9)	-	-
Missing	3 (0.5)	1 (0.1)	4 (0.3)		
**Employment**	605 (99.3)	798 (99.6)	1403 (99.5)		
Full time/part-time	519 (85.2)	498 (62.2)	1017 (72.1)	1,474,107 (74)^c^	7,203,234 (73)^c^
No paid work	86 (14.1)	300 (37.4)	386 (27.4)	507,319 (26)^c^	2,652,926 (27)^c^
Missing	4 (0.7)	3 (0.4)	7 (0.5)		
**Income**	570 (93.6)	769 (96.0)	1339 (95.0)		
<$52,000	170 (28.0)	239 (29.8)	409 (29.0)	569,175 (41)^c^	2,844,563 (41)^c^
≥$52,000	400 (65.6)	530 (66.2)	930 (66.0)	1,390,246 (59)^c^	6,935,474 (59)^c^
Missing	39 (6.4)	32 (4.0)	71 (5.0)		
**Country of birth**					
Australia	455 (74.7)	591 (73.8)	1046 (74.2)	47,190 (77.3)^a^	210,382 (71.4)^a^
Other	132 (21.7)	193 (24.1)	325 (23.0)	13,792 (22.6)^a^	82,740 (28.0)^a^
Missing	22 (3.6 )	17 (2.1)	39 (2.8)	38 (0.1)^a^	1,692 (0.6)^a^
**Aboriginal Torres Straits Islander**	12 ( 2.0)	15 (1.9)	27 (1.9)	3,504 (5.7)^a^	11,494 (3.9)^a^
Missing	-	2 (0.2)	2 (0.1)	-	792 (0.3)^a^
**BMI** Mean (SD, range)^a^ = Mean (n,%)	26.2 (5.9, 15.8 to 59.2)	27.0 (6.4, 13.4 to 78.4)	26.7 (6.2, 13.4 to 78.4)	25.9 (59,522, 97.5)^a^	26.0 (79,432, 82.4)^a^
<18.5 n (%)	10 (1.6)	11 (1.4)	29 (2.0)	2,625 (4.4)^a^	3,273 (4.1)^a^
18.5 – 24.9 n (%)	296 (48.6)	327 (40.8)	615 (43.6)	27,262 (45.8)^a^	36,488 (45.9)^a^
25.0 - 29.9 n (%)	154 (25.3)	221 (27.6)	375 (26.6)	16,419 (27.6)^a^	21,851 (27.5)^a^
>30 n (%)	110 (18.1)	190 (23.7)	300 (21.3)	13,216 (22.2)^a^	17,820 (22.4)^a^
Missing n (%)	39 (6.4)	52 (6.5)	91 (6.5)	1,498 (2.5)^a^	16,930 (17.6)^a^
**Obstetric factors**					
Nulliparous	609 (100)	-	609 (43.2)	24,876 (40.8)^a^	93,482 (42.1)^a^
Multiparous	-	801 (100)	801 (56.8)	36,144 (59.2)^a^	128,608 (57.9)^a^
**Characteristics of last birth**					
Non operative vaginal	-	-	522 (65.2)	35,071 (57.5)^a^	166,208 (56.4)^a^
Operative vaginal	-	-	78 (9.7)	5,892 ( 9.7)^a^	35,405 (12.0)^a^
**Caesarean section**	-	-	194 (24.2)	20,057 (32.9)^a^	93,157 (31.6)^a^
Labour/CS	-	-	131 (16.4)	7,450 (12.2)^a^	38,274 (13.0)^a^
No labour/CS	-	-	63 (7.9)	12,607 (20.7)^a^	54,876 (18.6)^a^
Missing	-	-	7 (0.9)	-	51 (0.0)^a^
Last Birth Pre-term	-	-	57 (7.1)	4,754 (7.8)^a^	22,004 (7.5)^a^
Last Birth Term	-	-	737 (92.1)	56,260 (92.2)^a^	272,760 (92.5)^a^
Missing	-	-	6 (0.8)	6 (0.0)^a^	50 (0.0)^a^
Live born	-	-	784 (97.9)	61,612 (98.9)^a^	297,357 (99.3)^a^
Stillborn	-	-	7 (0.87)	413 ( 0.7)^a^	2,206 (0.7)^a^
Neonatal death	-	-	3 (0.4)	235 (0.4)^a^	641 (0.2)^a^

^a^ [45]; ^b^ [46]; ^c^ [47]. State and National Employment figures pertain to all Australian women aged 16 to 49 [48].

### Obstetric factors

Just under half the women were nulliparous (n = 609, 43%) and 57% (n = 801) were multiparous. Of the multiparous women, 24% (n = 194) had a history of previous caesarean section (CS) in their last birth (emergency, n = 131, 16.%; elective, n = 63, 8%) (see Table 3). Compared to National and State data, women in the study had lower overall CS rates, but higher rates of emergency CS and lower rates of elective CS. Otherwise the study population had similar obstetric history to the general birthing population.

### Childbirth fear

Some 98% of women (n = 1,386) completed the WDEQ-A as a measure of childbirth fear. Of these, fear scores ranged from 0 to 128 (out of a possible total score of 165). The mean score was 49.5 (SD = 22) (refer to Table 4). Nearly 31% of women had low fear (37 or less) and 43% of women scored in the moderate fear range (38–65). Of the remaining women, 18.8% scored between 66–84 and 4.8% reported severe fear (85 or above). Refer Table 5.

**Table 4:** **Comparison of childbirth fear by demographic and obstetric factors**

Characteristics	WDEQ-A score (Md)	n (%)	Statistic	p-value	Effect
**Demographic**					
Age (WDEQ Mean)	49.5	1,382 (98.0)	r = -0.19	0.49	r = -0.19
In a relationship		1,380 (97.9)	z = -1.73	0.84	r = .05
Y	49	1,273 (90.3)			
N	56	107 (7.6)			
Education		1,382 (98.0)	t = .-.966	0.33	eta = -6.76
(WDEQ Mean)					
≤Yr12	48.9	691 (49.0)			
>Yr12	50.1	691 (49.0)			
Employment		1,379 (97.8)	z = -2.07	0.03	r = -0.06
Paid	50	1,000 (70.9)			
Unpaid	47	379 (26.9)			
Income		1,316 (93.3)	z = -.39	0.7	r = 0.01
<$78,000	50	775 (54.9)			
≥$78,000	50	541 (38.4)			
**Obstetric**					
Parity (WDEQ		1,386 (98.3)	t = 8.518	<0.001	eta = 0.05
Mean)					
Nullipara	55.1	604 (42.8)			
Multipara	45.2	782 (55.5)			
Last Birth Mode		775 (96.8)	z = -5.38	<0.001	r = -0.19
Non-operative	40	519 (64.8)			
Operative birth	51	256 (32.0)			
Missing		26 ( 3.2)

**Table 5:** **Level of childbirth fear by parity**

Parity	WDEQ-A ≤37 (low fear) n (%)	WDEQ-A 38–65 (moderate) n (%)	WDEQ-A 66–84 (high) n (%)	WDEQ-A >85 (severe) n (%)	Total n (%)
Nulliparous	126 (20.7)	288 (47.3)	151 (24.8)	39 (6.4)	604 (42.8)
Missing					5 (0.4)
Multiparous	309 (38.6)	330 (41.2)	114 (14.2)	29 (3.6)	782 (55.5)
Missing					19 (1.3)
Total	435 (30.9)	618 (43.8)	265 (18.8)	68 (4.8)	1386 (98.3)
Missing					24 ( 1.7)

### Childbirth fear and demographic characteristics

The only relationship found between demographic characteristics and childbirth fear were for women in paid work (Md = 50, n = 1,000) compared to those not in paid work (Md = 47, n = 379), U = 175826, z = -2.072, p = 0.038, r = .06). Refer to Table 4.

### Childbirth fear and parity

Analyses of fear levels (low, moderate, high and severe fear) by parity, are outlined in Table 5. An independent-samples t-test identified statistically significant higher WDEQ-A scores at baseline for nulliparous women compared to multiparous women (Mean = 55.05, SD = 20.7 vs. Mean = 45.18, SD = 21.9; t = (1,384) = 8.52, p <0.001, two tailed). Refer Table 4. This equated to nearly one in three (31.5%) women having their first baby scoring high (WDEQ-A ≥66) for childbirth fear compared to around one in five multiparous (18%) women (see Table 5).

### Childbirth fear, parity and last mode of birth

There was an association between childbirth fear, parity and last mode of birth. In comparing previous mode of birth for multiparous women, there were significant differences between women experiencing a normal vaginal birth (Md = 40, n = 519) compared with an instrumental birth (Md = 47, n = 77, U = 16474, z = -2.5, p = 0.013, r = -0.10); normal vaginal birth and emergency caesarean (Md = 53, n = 123, U = 23007, z = -4.82, p <0.001, r = -0.2), and normal vaginal birth and elective caesarean (Md = 52, n = 56, U = 11182, z = -2.84, p = 0.005, r = -0.12) (Table 4). Having a normal vaginal birth in a previous pregnancy was protective of childbirth fear in a subsequent pregnancy.

## Discussion

The current study recruited a large representative sample of Australian birthing women with baseline demographic characteristics similar to those of previous studies investigating prevalence of childbirth fear. Being in paid employment was the only demographic characteristic associated with childbirth fear. Two previous Swedish studies reported that women who were employed were more likely to receive treatment for childbirth fear [8, 49]. In our study, this might be explained by women having a first baby (who we found to have higher fear levels), more likely to be employed compared to women having subsequent babies and working in the home. Obstetric characteristics associated with childbirth fear were parity and previous birth mode.

We measured second trimester WDEQ-A scores. There is an assumption that childbirth fear may increase across pregnancy as anxiety is thought to be higher in third trimester [18, 50], and may be amenable to early intervention in the second trimester. However fear is not a stable construct [18, 37] and increasing levels of childbirth fear as pregnancy progresses has not been proven [37] and requires further research.

The prevalence of childbirth fear in our study aligns with the national and international literature. In Australia, UK, Canada and Sweden for the years 2001 to 2013 (inclusive of the current study), where populations have been assessed against WDEQ-A ≥66, prevalence of childbirth fear has been between 24% - 26% [6, 14, 16, 24]. Likewise, severe childbirth fear rates are comparable. Thirty years ago Areskog and colleagues [20] reported that around 17% women were ‘moderately’ fearful and a further 6% had ‘disabling’ fear. Our study found similar proportions (high fear 19%; severe fear 5%). When assessed against the higher fear score (WDEQ-A ≥85), prevalence has again been stable (8% - 11%) over a similar period of time and across countries (Table 1). Exceptions are two recent Swedish studies [27, 31] both citing prevalence for severe fear of 20%. This variation may be the result of both these studies having larger number of first time mothers.

In line with the findings of other researchers, especially where the WDEQ-A has been used as a screening tool, parity was statistically associated with childbirth fear [14, 28]. Nulliparous women had a 10-point higher mean WDEQ-A score compared to multiparous women. The reasons for higher fear levels as well as higher prevalence in women having their first baby may be related to the uncertainty of birth, or because these women are confronting a major life transition (physically, psychologically and socially) to becoming a mother. Some time ago Drummond and Rickwood [51] reported that women in a subsequent pregancy were less anxious than first time mothers as they had experienced birth before and consequently had knowledge that first time mothers had not yet acquired.

There is, however, some contradictory evidence around parity and fear. For example the early work of Zar et al. [24] reported slightly higher childbirth fear levels in multiparous women whereas Nieminen et al. [27] found no difference between parity groups. Similarly, the more recent work of Haines et al. [33] found no association between fear and parity in their Australian rural cohort. However in that study the Swedish comparison group did show a statistically significant association for giving birth for the first time and fear [33]. The different results may be attributable to fear being measured on a visual analogue scale (FOBs), which may be less sensitive than the WDEQ-A. While the FOBs may contribute to some variance, other social or environmental factors of living in a rural setting may contribute to differences. Rural women may have more exposure to normal childbirth, positive maternal role models, and birth stories or better social support.

Our results add to the growing body of evidence that demonstrates that normal vaginal birth is a protective factor for women in a subsequent pregnancy. When comparing women with experience of normal vaginal birth, and women with experience of any form of operative birth (forceps, vacuum, elective and emergency CS) high childbirth fear was more likely to be reported. Our results are consistent with recent research conducted by US researchers Elvander et al. [40] who found level of fear and birth outcome were related. The association between operative birth and high fear levels were utmost for both elective and emergency CS in the current study, although emergency CS was the most significant contributor to high childbirth fear scores. This is consistent with the findings of Swedish researchers Nilsson and colleagues [52] who reported that aside from the birth experience, emergency CS contributed most to fear levels. As Hildingsson et al. [53] argued, planning a CS will not cure women’s fear. Maternity health care professionals need to spend time eliciting women’s childbirth expectations, listening to their previous birth stories and engaging in positive birth planning. This is especially important for women that have experienced operative birth and those who have experienced a previous birth as traumatic. Where women are supported to achieve vaginal birth despite an initial request for CS, they are at least equally satisfied as those women who always intended a normal birth [54].

### Strengths and Limitations

While a strength of the current study was the use of the WDEQ-A, the most widely used tool for identifying childbirth fear [21], the use of other scales such as the brief scale by Areskog [55] or visual analogue scales [28, 33] make comparison of findings amongst all studies somewhat difficult. Comparing results is also hampered by variations in cut-off scores used for study recruitment and interpreting change. The current study, however, applied the original scores used with the WDEQ-A for defining high childbirth fear [24].

In addition, translation of tools to other languages may introduce variation in interpretation and detection of childbirth fear [14, 56]. Indeed in the current study several questions in the WDEQ-A were modified as the originally translated Swedish English versions of some words were not applicable to Australian women. Furthermore, women planning a CS had difficulty completing the WDEQ-A when asked questions pertaining, for example; to the intensity of labour. More recently the antenatal WDEQ-A and a postnatal version have been adapted by the original authors for use with women specifically planning a CS, but these adapted versions were not available at commencement of the current study. The original tool may have been off-putting to women planning a CS and may have prompted them to withdraw. High dropout rates in a previous study were attributed to the WDEQ-A not considering the needs of women planning CS [36]. Our results also need to be considered in light of the recruitment of women from public hospital clinics which may underestimate prevalence as one third of Australian birthing women receive private obstetric care.

## Conclusion

The current study recruited a large representative sample of Australian birthing women. The prevalence of high childbirth fear in Australian women was comparable to international studies when measured using the same validated tool, and indicates childbirth fear is a significant factor for women’s emotional wellbeing during pregnancy. While limited demographic characteristics were associated with childbirth fear, obstetric indicators of parity and mode of last birth were significant. Nulliparity was a predictor for childbirth fear. For multiparous women, experiencing an operative birth was statistically associated with childbirth fear in the next pregnancy. Birth mode is a readily identified risk factor for women reporting fear in a subsequent pregnancy. Best practice should involve determining the specific concerns of every woman to enable health care providers to address and alleviate these worries. In addition, these findings support the importance of keeping birth normal in a first pregnancy. Further investigation of psychosocial characteristics that may be amenable to change and their association with childbirth fear are now warranted.

### Human Research Ethics Committee approval numbers

HREC/11/QGC/162 (Queensland Health); NRS/45/11/HREC (Griffith University).

## Abbreviations

FOBs: Fear of birth scale
CS: Caesarean section
UK: United Kingdom
US: United States
WDEQ-A: Wijma delivery expectancy/experience questionnaire version A.
